# Effects of the incorporation of honey from *Apis mellifera* from the Amazon savanna on the technological and sensory properties of yogurt during refrigerated storage

**DOI:** 10.1371/journal.pone.0344493

**Published:** 2026-03-17

**Authors:** Manoel Henrique do Rosário Silva, Maria Clarisnete de Oliveira Moura, Esther Morais da Silva Assuncão, Kathielle Delfraxe Santos Oliveira, Mateus Lima Ramos, Adria Kemilly Costa Moura, Ana Paula Folmer Correa, Laura Adriane de Moraes Pinto, Daniela Cavalcante dos Santos Campos, Gardenia Holanda Cabral, Marcos Jose Salgado Vital, Jéssica de Oliveira Monteschio

**Affiliations:** 1 Postgraduate Program in Natural Resources, PRONAT, Federal University of Roraima, Boa Vista, Roraima, Brazil; 2 Postgraduate Program in Biodiversity and Biotechnology, Bionorte, State Coordination of Roraima, Federal University of Roraima, Boa Vista, Roraima, Brazil; 3 Agrarian and Environmental Sciences Institute, Federal University of Mato Grosso, Sinop, Mato Grosso, Brazil; 4 Department of Food Engineering, State University of Middle West, Guarapuava, Paraná, Brazil; 5 Agrotechnical School of Federal University of Roraima, Campus Murupu, Boa Vista, Roraima, Brazil; Lusofona University of Humanities and Technologies: Universidade Lusofona de Humanidades e Tecnologias, PORTUGAL

## Abstract

The development of new yogurt formulations that provide additional health benefits has gained prominence in recent years. The objective of this study was to characterize the physicochemical, antioxidant, technological, microstructural and sensory properties of the honey of *Apis mellifera* bees from the Amazon savanna and evaluate its inclusion at different concentrations (8%, 12%, 16% and 20%) of yogurt during 28 days of refrigerated storage. The results showed that honey is within the recommended quality parameters for this product. The amount of added honey had positive effects on the color characteristics, water holding capacity and total solids content (P < 0.05), increasing the nutritional value of the yogurt. The highest concentrations of honey (16% and 20%) presented relatively high antioxidant activity (measured via the DPPH method) and were the most sensorially accepted (P < 0.05) by consumers. In addition, the highest concentration of honey (20%) favored the stability of syneresis and resulted in higher levels of total polyphenol compounds (P < 0.05). In conclusion, the addition of honey from *Apis mellifera* from the Amazon savanna to yogurt formulation proved to be an effective strategy to improve the quality and acceptability of the final product. In addition to standing out as a promising ingredient for the development of functional foods, its incorporation represents a relevant innovation for the local food sector, with new perspectives for the creation of differentiated products aligned with the contemporary demands for healthy and sustainable foods.

## Introduction

In recent years, consumer demand for foods with a balanced nutritional composition and additional health benefits has increased significantly [1]. Foods with functional properties that are capable of providing essential nutrients while also contributing to disease prevention and improved quality of life have become particularly valued [2,3].

Ingredients with bioactive potential have been incorporated into various formulations with the aim of enhancing products and adding functional attributes. Among these foods, fermented milk products such as curds, dairy beverages, and yogurt stand out as promising matrices for the development of functional foods [3].

With fermented dairy products, yogurt stands out for its beneficial properties, such as antimicrobial activity, stimulation of the immune system and improvement in the digestibility of proteins and lipids [2,4,5].

Furthermore, yogurt is a versatile food that can be consumed in its natural form or enriched with flavors and sweeteners for the development of new formulations [6,7].

Regardless of age group, consumers tend to prefer foods with a relatively high sugar content [8–10]. However, although yogurt is widely recognized as a healthy food, this positive perception can be compromised by the use of artificial ingredients, such as synthetic sweeteners [8,9,11].

Since the health benefits of a dairy product cannot compensate for shortcomings in its sensory attributes and since sensory acceptance is a decisive determinant of consumer preference, the use of natural sweeteners has emerged as a promising strategy to enhance flavor while simultaneously adding functional value to the product [9].

Honey from *Apis mellifera* honey is widely valued for its sweetening ability, pleasant taste, aroma and nutritional composition, which is rich in vitamins, minerals and small amounts of protein [3,12]. Furthermore, its matrix also contains oligosaccharides with prebiotic effects that are capable of stimulating the growth of beneficial microorganisms in the gut and promoting digestive health [2,13]. From a functional point of view, honey is recognized as a significant source of bioactive compounds, especially polyphenols and other phenolics, which exhibit high antioxidant activity [3,5,14,15]. These metabolites have been extensively investigated for their anti-inflammatory and antimicrobial properties, contributing to the relevance of honey as a functional ingredient [16–18].

Brazil is one of the countries with the greatest diversity of honeys in the world, a direct result of the wide variety of ecosystems, native flora, and tropical and subtropical climatic conditions [19]. Monofloral and wild honeys are produced in biomes such as the Amazon, Cerrado, Atlantic Forest, Caatinga, and Pampa, each conferring unique characteristics in terms of color, aroma, viscosity, and phenolic profile. [20–22].

In the Amazon, in particular, a hot and humid climate combined with high plant biodiversity favors the production of wild honeys with high sensory complexity and high levels of antioxidant compounds [23,20]. The abundant flowering that occurs after the rainy season contributes to high-quality honeys, which are often valued for their functional properties and nutraceutical potential [21,22,20,24]. This diversity makes Brazil an important beekeeping hub, with products capable of adding value to different food matrices.

The phenolic compounds present in honey are biologically active secondary metabolites of plants with potent natural antioxidants [25]. These compounds exert protective effects by neutralizing or reducing the formation of free radicals, which are associated with oxidative stress and the development of various chronic diseases [26,27]. In addition, polyphenols modulate inflammatory processes, reinforcing the role of honey as a functional food with potential health benefits [1,27].

Although the beneficial properties of honey are widely recognized, the potential of honey from the Amazon savanna within complex food matrices, such as yogurt, and its influence during storage have not yet been investigated.

The objective of this study was to characterize Amazonian savanna honey and evaluate the impact of its incorporation at different concentrations (8–20%) on the physicochemical, antioxidant, technological, microstructural, and sensory properties of yogurt stored for 28 days.

## Materials and methods

### Obtaining the ingredients

Commercial whole cow milk with ultrahigh heat treatment (UHT) reconstituted with 3% fat (Italac®, Goiás, Brazil), skim milk powder consisting of 0.5% fat (Italac®, Goiás, Brazil) and crystal sugar (União®, Rio de Janeiro, Brazil) were obtained from the local market. The thermophilic freeze-dried dairy culture was obtained from the company Rica Nata® (Ricaferm YR03 – *Streptococcus Salivarius subsp. thermophilicus, Lactobacillus delbrueckii subsp. Bulgaricus*, Piracema, Minas Gerais, Brazil). Honey from *Apis mellifera* bees from the Amazon savanna was purchased from the Northern Beekeeping Association of Roraima – Processing Unit of Bee Producers (Roraima, Boa Vista, Brazil). The analyses were performed between January and November 2024 with three honey batches. After each handling event, the physicochemical parameters were reassessed to guarantee analytical homogeneity and preserve sample integrity.

### Honey characterization

The characterization of honey was performed by evaluating the parameters of moisture, acidity, hydroxymethylfurfural (HMF), Brix and pH according to the methods described by the Association of Official Analytical Chemists [28].

Color determination was performed via spectrometry, and the results were compared with those of the Pfund color scale [29]. The samples with values ≤ 0.44 nm were classified as light honey, and those with values > 0.44 nm were classified as dark honey.

To evaluate the antioxidant activity (DPHH) and phenolic compounds (TPC), honey was previously diluted in distilled water at a ratio of 1:5 (w/v). The analysis of total phenolic content (TPC) was performed via the Folin‒Ciocalteu method, as described previously by Gomes et al. [20], with modifications.

Aliquots of aqueous honey solutions (0.2 g/mL) were homogenized with 905 µL of distilled water, 120 µL of Folin–Ciocalteu reagent and 800 µL of sodium carbonate solution (15%). The mixture was analyzed on polystyrene microplates, and the absorption was measured at 798 nm. The results are expressed as mg of gallic acid equivalent (GAE) 100 g ^−1^.

The free radical scavenging activity of DPPH was determined as described by Silva et al. [30], with modifications. An aliquot of 0.2 mL of honey mixture was diluted in 0.2 mL of methanol and 1.6 mL of DPPH solution. The mixture was kept in the dark at room temperature for 30 minutes. The absorbance was measured with a spectrophotometer at 515 nm, and the DPPH radical scavenging capacity was expressed as a percentage.

### Yogurt preparation

The cow milk used in the preparation of the yogurt was initially analyzed for nonfat solids (8.1%), fat (3.0%), protein (2.9%), density (29.8%), lactose (4.4%), mineral salts (0.6%), freezing point (−0.515 °C), added water (0.0%), pH (6.74) and conductivity (5.5) via a Master Classic portable milk analyzer obtained from Ismart® (Santa Maria, São Paulo, Brazil).

To prepare the yogurt, whole cow milk was added to powdered skim milk (4%). The mixture was homogenized and pasteurized at 85 °C for 30 minutes. After thermal treatment, the milk was cooled to 43 °C and inoculated with a commercial culture containing *Streptococcus salivarius* subsp*. thermophilicus and Lactobacillus delbrueckii* subsp*. bulgaricus*, following the manufacturer’s recommendations. The mixture was fermented in a temperature-controlled incubator (43 °C) until the pH reached 4.6, as described by Jiménez-Redondo et al. [7]. The yogurt was subsequently cooled to 4 °C, and the clots were broken by manual agitation with a glass rod.

Then, the mixture was gently homogenized and flavored (sugars or honey). In the control treatment, without the addition of honey, 5% sucrose was added. Different concentrations of bee honey (*A. mellifera* from the Amazon savanna) were added to the experimental treatments: H8% (yogurt with the addition of 8% honey), H12% (yogurt with the addition of 12% honey), H16% (yogurt with the addition of 16% honey) and H20% (yogurt with the addition of 20% honey). The selection of honey concentrations was based on previous evidence of sensory acceptance among Brazilian consumers, as well as the need to evaluate potential functional benefits associated with incorporating honey into yogurt [6].

The yogurt samples were placed in high-density polyethylene bottles, capped and stored under refrigeration (4 ± 2 °C). The analyses of the physical-chemical, technological and functional properties were conducted with eight repetitions per treatment in each analysis performed on days 1, 3, 7, 14, 21 and 28 of cold storage. Sensory and microstructure analyses of the yogurt were performed exclusively on the first day of refrigerated storage.

### Physicochemical parameters of yogurt

#### pH, titratable acidity, total solids and mineral content.

Changes in pH during fermentation and storage were monitored via a portable digital pH meter previously calibrated with buffers 4 and 7 (Testo – 205).

The titratable acidity was determined according to the methodology proposed by Vital et al. [31]. The yogurt was mixed with distilled water (1:1 w/v) and titrated with 0.1 M sodium hydroxide solution to pH 8.3 ± 0.01. The titratable acidity was expressed as g lactic acid/100 g yogurt and calculated via equation (1):


$$\text{Titratable acidity }(\text{g}/\text{100g})\;=\frac{\text{ V x f x }\text{0}.\text{9}}{\text{m}}\;$$ (1)

where V is the volume (mL) of 0.1 M sodium hydroxide solution used in the titration; m is the mass (g) of the test sample; 0.9 is the conversion factor for lactic acid; and f is the molarity of the sodium hydroxide solution [32].

The total solids content and ash content of the yogurt were determined according to the methods of the Association of Official Analytical Chemists [28].

### Antioxidant activity (DPPH radical scavenging) and total phenolic compounds (TPC)

The extracts were obtained by extracting yogurt with ethanol (1:1 w/v with ethanol) followed by centrifugation (4 °C and 11000 × g for 30 min) and qualitative filtration. The extract was stored at −20 °C until the analyses were performed to determine phenolic compounds and antioxidant activity [33].

DPPH radical scavenging activity was measured according to Monteschio et al. [34]. The yogurt extract was mixed with a methanolic DPPH solution (60 μM) and incubated for 30 minutes in the dark. The absorbance was measured at 515 nm in a plate reader (Synergy HT, Biotek). The absorbance (A) at 515 nm was measured against a blank of pure 95% ethanol at t = 0. The antioxidant activity was calculated via equation 2:


$$\begin{array}{c} \%\text{ DPPH scavenging activity }=\;(\text{1}-\;(\frac{\text{A sample }}{\text{A control t0}})\;\times \text{ 100} \end{array}$$ (2)

where a control _t0_ is the absorbance of the control sample (blank of pure 95% ethanol) at time 0 and A_sample_ is the absorbance of the sample at 30 min.

TPC analysis was performed via the Folin‒Ciocalteu method, as described by Affonso et al. [35]. To determine the TPC content, an aliquot of 0.25 mL of the extract obtained above was mixed with 0.25 mL of the Folin–Ciocalteu reagent and 2 mL of distilled water. After 3 minutes at room temperature, 0.25 mL of a saturated solution of sodium carbonate was added, and the mixture was placed in a water bath at 37 °C for 30 minutes. The absorbance was measured at 750 nm via an Ultrospec 2000 UV/visible spectrophotometer (Amersham Biosciences, Cambridge, UK). A calibration curve of gallic acid was used as a reference standard (R² = 0.995), and the results are expressed as mg of gallic acid equivalents (GAE) per 100 g of sample in fresh weight (FW) [36].

### Technological properties

#### Color, syneresis and determination of the water holding capacity (WHC).

The CIELab color parameters were recorded via a calorimeter (Minolta, Japan) under D65 lighting with a viewing angle of 10°, adjusted to the L * a * b * system. Six measurements at random points were recorded per sample, obtaining luminosity (L *), redness (a *), and yellowing (b *) [37].

To evaluate the syneresis of the yogurt, 10 g of the sample was placed in a funnel lined with paper number 1 (Whatman International Ltd., Maidstone, UK). The volume of serum collected was placed in a beaker, and the degree of syneresis was calculated according to Gölbaşι et al. [38] (Eq 3).


$$\text{Syneresis }(\%)\;=\;\frac{\left(\text{Weight of whey collected after drainage }\right)}{\text{Weight of yogurt sample}}\;\times \text{1}\text{0}\text{0}$$ (3)

The water holding capacity (WR) of the yogurt was determined according to the method of Nazari et al. [39]. Ten grams of yogurt was centrifuged at 6500 rpm (4 °C for 15 min). After centrifugation, the whey layer was removed, and the final weight was recorded. The WRC of the yogurt was determined according to equation (4).


$$WRC\;(\%)=\frac{\left(Weight\;of\;yogurt\;sample\;-wheight\;of\;whey\right)}{Weight\;of\;yogurt\;sample}\times \text{1}\text{0}\text{0}$$ (4)

### Microstructure

The microstructure was determined as described by Vital et al. [31]. The yogurt samples were frozen in nitrogen and freeze-dried to preserve their structure. The samples were mounted on aluminum stubs and coated with a layer of gold (Spotter coating, Baltic, SCD 050). The samples were analyzed by scanning electron microscopy (SEM) at 10 kV.

### Sensory analysis

The sensory analysis study was approved by the Research Ethics Committee for Studies Involving Human Beings of the Federal University of Roraima (protocol number 69320823.2.0000.5302, approved on March 20, 2024), Boa Vista, Roraima, Brazil.

Participant recruitment was conducted prospectively on November 24, 2024, at the Cauamé Campus of the Federal University of Roraima. All participants were over 18 years of age, were fully informed about the objectives and procedures of the study, and provided written informed consent prior to participation. No minors were included in the study, ensuring full compliance with ethical guidelines for research involving human subjects.

The sensory evaluation of the yogurt was performed in individual cabins with controlled temperature and lighting conditions. The untrained consumers were randomly selected (students and employees of the Federal University of Roraima). Each consumer evaluated five samples, one per treatment (CON, H8%, H12%, H16% and H20%), in a white cup coded with 3-digit numbers and served at random.

All the consumers evaluated the acceptability of yogurt for the following attributes: appearance, color, aroma, flavor, texture and general acceptability, which were evaluated on a nine-point structured hedonic scale (1 = dislike extremely; 9=like extremely), without the level of medium, according to de Oliveira Monteschio et al. [40].

Purchase intention and consumer preference were evaluated via structured five-point scales ranging from 1 (lowest intensity) to 5 (highest intensity). For purchase intention, 1 corresponded to “definitely would not buy” and 5 to “definitely would buy,” whereas for preference, 1 indicated “least preferred” and 5 the “most preferred,” according to the method described by Machado et al. [6].

### Statistical analysis

Statistical analyses were performed via SPSS Statistics software (version 23.0; IBM Corp., Armonk, NY, USA) for Windows. A completely randomized factorial design was implemented with five treatments (control, 8%, 12%, 16%, and 20% honey) and six storage periods (1, 3, 7, 14, 21, and 28 days). The response variables were subjected to analysis of variance (ANOVA) to evaluate the main effects of treatment and storage time, as well as their interaction. When significant differences were detected, the means were compared via Tukey’s test at the 5% significance level (p < 0.05).

The results are expressed as the means of eight independent replicates, each performed in triplicate for every experimental condition. For the consumer sensory analysis, the experimental design considered treatment (honey concentration) as a fixed effect and consumer as a random effect. The response variable was the sensory acceptability of the samples.

## Results and discussion

### Honey characterization

The results of the characterization analyses of honey from *Apis mellifera* bees from the Amazon savanna were compared with the limits established by Normative Instruction n°11 of the Ministry of Agriculture and Food Supply [41] and are in accordance with legislation (Table 1).

**Table 1:** Characterization of honey from *Apis mellifera* bees from the Amazon savanna.

Analysis	Regulation^1^	Honey	SEM^2^
Color absorbance	–	0.20	0.000
Free acidity^3^	< 50	25.95	0.004
pH	–	3.64	0.005
Moisture^4^	< 20	17.97	0.005
Minerals (ash)^4^	< 0.6	0.29	0.005
Apparent sucrose^4^	< 6	2.86	0.010
Reducing Sugars^4^	> 65	73.63	0.010
Water-insoluble solids^4^	< 0.1	0.04	0.005
Soluble solids^5^	–	80.30	0.005
Diastatic activity^6^	> 8	8.7	0.050
Hydroxymethylfurfural^7^	< 60	17.47	0.005
DPPH^8^	–	46.07	0.514
TPC^9^	–	19.02	0.348

^**1**^Normative Instruction n°11 of the Ministry of Agriculture and Food Supply; ^**2**^SEM: Standard error of means; ^3^Unit expressed in Meq/kg of honey; ^4^Unit expressed in g/100 g of honey; ^5^Unit expressed in °Brix; ^6^Diastasis activity measured by the Gothe Scale; ^7^Unit: mg Hydroxymethylfurfural/kg of honey; ^8^DPPH – % DPPH radical scavenging activity; ^9^TPC - Total phenolic compounds expressed in mg gallic acid equivalents (GAE)/100 g of honey.

The absorbance value at 0.201 nm was used to classify honey into “extra light amber” and “light amber” categories according to Góes Nascimento & Benevides [42]. Color is an important factor in the acceptability of honey, with lighter honeys being preferred on the market and having greater commercial value [42–44].

The free acidity and pH values remained within regulatory limits, indicating the absence of fermentation or adulteration, conditions that typically alter honey pH [45,46]. The acidity observed reflects the presence of organic acids inherent to different nectar sources, the action of glucose oxidase, and the contribution of minerals naturally present in honey [47].

The moisture content also complied with current legislation [48,49], and the ash content (0.29 g/100 g) was below the regulatory limit (≤ 0.60 g/100 g), indicating proper handling and the absence of mineral contamination resulting from inadequate extraction, decanting, or filtration practices [50]. Taken together, these parameters confirm that the product is chemically stable and properly preserved. Similar results for pH, moisture, total acidity, and ash have been reported by Farias et al. [51] for honeys from the Amazonian Cerrado, suggesting a consistent regional pattern.

The sucrose content observed, within regulatory limits, indicates that harvest occurred at an appropriate stage, as elevated concentrations of this sugar generally suggest premature collection when the conversion of sucrose into glucose has not yet been completed [52,47]. In the analyzed samples, the predominant sugars were fructose (38%) and glucose (31%), both of which play important functional roles, fructose due to its high hygroscopicity, and glucose due to its direct influence on crystallization [53,54].

The concentration of insoluble solids remained within the expected standards, reflecting low levels of wax residues, bee fragments, and other particles derived from extraction and processing. These results reinforce the adoption of good hygienic practices and adequate technological control [30,45]. The content of soluble solids (°Brix) was high, as expected, given that honey contains approximately 80% sugars [55,56]. The sucrose content remained within the permitted limit.

The sugar profile and solid content, when considered together with pH, acidity, and moisture, indicate mature, concentrated, and stable honey, suggesting harvesting at the proper time, as elevated concentrations indicate premature collection and incomplete conversion of sucrose into glucose and fructose [47,52].

The diastase activity (8.7 on the Göethe scale) and hydroxymethylfurfural (HMF) levels met the minimum legal requirements, indicating an acceptable degree of preservation. However, values close to the regulatory threshold suggest partial loss of enzymatic activity, potentially associated with storage duration or exposure to heat during processing [57]. Elevated HMF is related to overheating or inadequate storage conditions [58]. Although the product remains legally compliant, this finding underscores the importance of rigorous monitoring of handling and storage conditions to preserve enzymatic integrity [46,50,59].

The antioxidant activity, measured by DPPH radical scavenging (46.07%), fell within the range reported for Amazonian honeys and was similar to values described by several authors [20,54,60].

This antioxidant potential is attributed to phenolic compounds, phenolic acids, and free amino acids [60,61], which contribute to reducing oxidative stress and provide potential health benefits [18,62,63].

The total phenolic content (19.02 mg GAE/100 g) was also consistent with values reported for honeys from southern and northern Brazil [19,64] and is largely influenced by botanical origin [65]. Importantly, although this method is widely used, it may also respond to other reducing compounds present in complex food matrices, such as sugars and proteins [66].

Previous studies have shown that darker honeys, such as the one evaluated in this study, tend to exhibit higher phenolic concentrations and, consequently, greater antioxidant activity [19,67].

Phenolic compounds are known to establish noncovalent interactions with milk proteins, including caseins and whey proteins, mainly through hydrogen bonding and hydrophobic interactions [68]. These interactions may alter the three-dimensional configuration of proteins, reinforcing the cohesion of the gel network and resulting in greater stability and improved texture of the final product [14,69,70]. In addition, phenolic compounds may increase the oxidative stability of the dairy matrix by providing antioxidant capacity against free radicals generated during processing and storage.

The moderate total phenolic content and scavenging activity observed may reflect differences in nectar sources and soil mineral composition, which directly influence secondary metabolite synthesis in plants [56].

Nevertheless, it is important to emphasize that Amazon savanna honey is strongly influenced by regional botanical diversity, which affects phenolic composition, the sugar profile, enzyme activity, and antioxidant capacity.

### Physicochemical parameters of yogurt

#### pH, titratable acidity, total solids and mineral content.

The pH values of the yogurt samples differed among treatments and across storage times (P < 0.05) (Table 2). The formulations containing higher honey concentrations (12%, 16%, and 20%) presented significantly lower pH values than did the control (CON) and the groups treated with 8% honey (H8%). The reduction in pH was proportional to the amount of honey incorporated into the formulation. Throughout storage, a progressive decrease in pH was observed up to day 28 (P < 0,05). When both factors were evaluated jointly, no interaction effect was detected. This pattern may be associated with the ability of honey to stimulate the metabolic activity of lactic acid bacteria, thereby enhancing acidification of the formulations [6,71]. In addition, the natural acidity of honey and the presence of fermentable carbohydrates, such as glucose and fructose, as well as organic acids inherent to its composition, may have further contributed to the observed pH reduction [72].

**Table 2:** Physicochemical parameters of yogurt with and without the addition of bee honey *A. mellifera* from the Amazon savanna at different concentrations over 28 days of refrigerated storage.

	Treatments					Display							P < Value		
Analysis	CON^1^	H8%^2^	H12%^3^	H16%^4^	H20%^5^	1	3	7	14	21	28	SEM^6^	T^7^	D^8^	TxD^9^
pH	4.55^a^	4.49^b^	4.45^c^	4.44^c^	4.42^c^	4.55^A^	4.52^AB^	4.50^B^	4.49^B^	4.40^C^	4.37^C^	0.009	0.000	0.000	0.456
TA^10^	1.00^bc^	0.99^c^	1.01^bc^	1.02^b^	1.07^a^	0.82^D^	1.03^C^	1.03^C^	1.06^B^	1.07^AB^	1.09^A^	0.010	0.000	0.000	0.000
TS^11^	21.31^e^	23.34^d^	25.03^c^	25.51^b^	26.77^a^	24.66^A^	24.64^AB^	24.33^ABC^	24.30^C^	24.31^BC^	24.11^C^	0.204	0.000	0.000	0.098
FMR^12^	1.56	1.41	1.34	1.36	1.32	1.40	1.40	1.42	1.35	1.40	1.40	0.041	0.528	0.999	0.990

Means of treatments with different lower-case letters in the same line are significantly different (P < 0.05). The means of storage with different uppercase letters are significantly different (P < 0.05).^1^CON– yogurt without bee honey; ^2^H8% - yogurt containing bee honey at 8% (v/v); ^3^H12% - yogurt containing bee honey at 12% (v/v); ^4^H16% - yogurt containing bee honey at 16% (v/v); ^5^H20% – yogurt containing bee honey at 20% (v/v). ^6^SEM: Standard error of means; ^7^T = P value effect of treatment; ^8^D = P value effect days of storage; ^9^TxD = P value interaction between treatments and storage.^10^TA: Titratable acidity (g lactic acid/100 g);^11^TS: Total solids (g/100 g); ^12^FMR- Fixed mineral residue (g/100 g).

In a study conducted by Ammar et al. [73] The pH of yogurt containing honey and probiotic cultures (*S. thermophilus*, *Lb. acidophilus* and *B. bifidum*) was lower than the pH of yogurt containing only probiotic cultures, both at the beginning and at the end of storage (15 days), which was also observed in our study.

**Fig 1:**
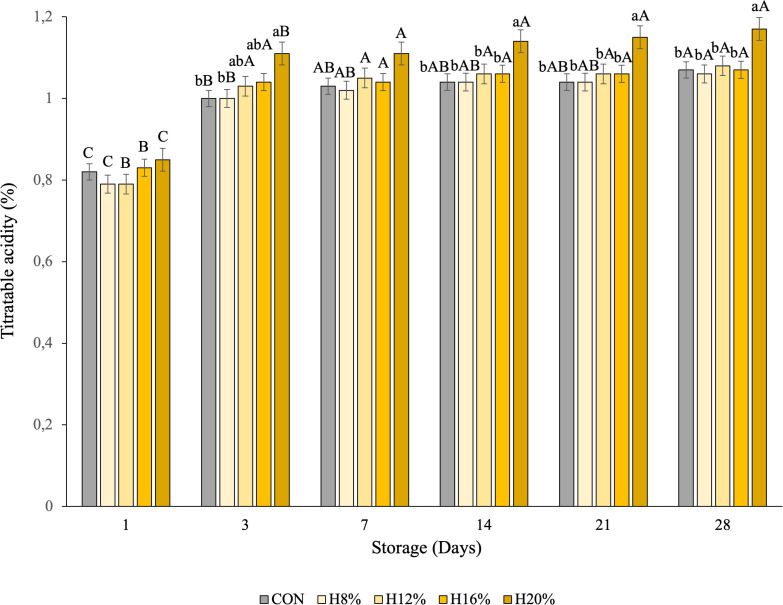
Interaction effects of treatment and days of storage on the titratable acidity of yogurt with honey during storage. Different lower-case letters indicate significant differences between treatments on the same day of display (P < 0.050). Different uppercase letters indicate significant differences in the same treatment (P < 0.050).^1^CON– yogurt without bee honey; ^2^H8% - yogurt containing bee honey at 8% (v/v); ^3^H12% - yogurt containing bee honey at 12% (v/v); ^4^H16% - yogurt containing bee honey at 16% (v/v); ^5^H20% – yogurt containing bee honey at 20% (v/v).

The total titratable acidity of the yogurt varied significantly between treatments and throughout storage (P < 0.05), with values of titratable acidity between 1.00 and 1.07 g lactic acid/100 g. These values are within the limits established by Brazilian legislation (0.6 to 1.5 g lactic acid/100 g) [74]. An interaction was observed between the effects (treatments and storage time), as shown in Fig 1.

On the 3rd day of storage, the CON and H8% yogurt samples had lower acidity than did the H20% honey samples. On the 14th day, the acidity of yogurt enriched with H20 was greater than that of yogurt supplemented with the other formulations. An increase in acidity over the storage period was observed in all the yogurt formulations evaluated (P > 0.05).

This increase may be associated with the presence of prebiotic oligosaccharides in honey, which, even in small amounts, can promote the growth and/or metabolic activity of lactic acid bacteria, as previously reported by Machado et al. [6]. In addition, the increase in acidity during storage may be associated with the release of water (syneresis) in these samples, resulting from protein denaturation, which occurs when the pH reaches the isoelectric point of the proteins, destabilizing the casein micelles and causing the loss of water liquid [6,75], which was also observed in our study.

Previous studies, such as that of Ammar et al. [73], reported that the titratable acidity of yogurt with acacia honey was greater at the beginning and on the last day of storage (15 days) than that of yogurt without honey. Moreira et al. [76], when evaluating the quality of apple yogurt sweetened with sucrose and honey, reported a total titratable acidity of 0.68% in apple yogurt sweetened with honey.

The total solids content of yogurt significantly increased with the addition of honey (P < 0.05). As the proportion of honey in the formulation increased, the total solids content rose accordingly. The highest total solids concentration was observed in the treatment containing 20% honey (Table 2). *Apis mellifera* honey is naturally rich in sugars and other solid compounds, such as proteins, minerals and vitamins, making it rich in macronutrients, which may have contributed to this behavior [3,50].

Higher concentrations of total solids contribute to an enhanced nutritional profile, resulting from a higher concentration of nutrients. Furthermore, they favor improvements in the functional properties of a product, such as its antioxidant capacity, due to the greater presence of bioactive compounds, especially polyphenols, which are recognized for their potential health benefits [77].

During storage, a significant reduction (P ≤ 0.05) in the total solids content of the formulations was observed at the end of 28 days. This behavior can be explained by structural changes in the protein matrix, such as progressive denaturation, rearrangement of the gel network, and increased hydration, which increase the solubility of solid fractions in whey over time [78]. Furthermore, the redistribution of water between the protein matrix and sugars due to osmotic gradients also contributes to this effect [79]. Although honey initially attracts water due to its high hygroscopicity, subsequent re-equilibration between bound and free water occurs, which can result in a state distinct from that observed at the beginning of storage [68,80]. When both factors were evaluated simultaneously, no interaction was detected.

A similar result was reported by Sert et al. [81], who evaluated the influence of adding sunflower honey (2%, 4% and 6% w/v) on yogurt preparation during refrigerated storage, and by Ismail et al. [82], who studied the addition of honey in the elaboration of functional dairy products with goat milk. The total dry extract contents of the yogurt significantly differed (P < 0.05) during refrigerated storage, with a decrease compared with the initial time. This behavior was also reported by Feitosa et al. [3], who evaluated the physicochemical stability of yogurt sweetened with *Apis mellifera* L.

With respect to the mineral content, no significant differences were observed between the treatments and storage days (P > 0.05) (Table 2). The combined evaluation of both effects revealed no significant interaction.

### Yogurt antioxidant activity

Yogurts formulated with relatively high honey concentrations (H16% and H20%) presented greater DPPH radical-scavenging activity (P < 0.05) than did the control yogurt (CON, without honey) and yogurt supplemented with low amounts of honey (H8%) (Table 3).

**Table 3:** Antioxidant activity of yogurt with and without added honey *A. mellifera* from the Amazon savanna at different concentrations during 28 days of refrigerated storage.

	Treatments					Display							P < Value		
Analysis	CON^1^	H8%^2^	H12%^3^	H16%^4^	H2O%^5^	1	3	7	14	21	28	SEM^6^	T^7^	D^8^	TxD^9^
DPPH^10^	42.04^b^	42.93^b^	47.39^ab^	49.30^a^	49.99^a^	50.07^A^	48.33^A^	46.71^AB^	46.41^AB^	45.59^AB^	40.40^B^	0.748	0.000	0.004	0.983
TPC^11^	35.90^c^	39.61^bc^	40.02^bc^	44.79^ab^	47.42^a^	66.52^A^	38.57^B^	36.12^BC^	34.33^BC^	32.10^C^	33.12^C^	1.742	0.000	0.000	0.957

Means of treatments with different lower-case letters in the same line are significantly different (P < 0.05). The means of storage with different uppercase letters are significantly different (P < 0.05).^1^CON – yogurt without bee honey; ^2^H8% – yogurt containing bee honey at 8% (v/v); ^3^H12% - yogurt containing bee honey at 12% (v/v); ^4^H16% - yogurt containing bee honey at 16% (v/v); ^5^H20% – yogurt containing bee honey at 20% (v/v). ^6^SEM: standard error of means; ^7^T = P value effect of treatment; ^8^D = P value effect days of storage; ^9^TxD = P value interaction between treatments and storage; ^10^DPPH – % DPPH radical scavenging activity; ^11^TPC- total phenolic compounds expressed in mg gallic acid equivalents (GAE)/100 g of yogurt.

The H20% formulation also presented the highest total phenolic content (TPC), confirming its superior antioxidant effect relative to the other treatments, including the control. These results are directly associated with the presence of bioactive compounds in honey, especially polyphenols, which are known for their antioxidant and antibacterial properties [4,27,72,83], as evidenced in the initial characterization of honey (Table 1).

When the initial and final antioxidant contents of the yogurt samples were compared, the antioxidant activity measured by DPPH remained within a similar range over time, whereas the TPC values increased at the end of storage. This pattern may be related to the low specificity of the Folin‒Ciocalteu (FC) assay, which measures the overall reducing capacity of the sample rather than exclusively phenolic compounds for honey, due to interference from reducing sugars or even interactions with proteins and amino acids, such as tyrosine and tryptophan, in yogurt, which can react strongly with the FC reagent, potentially leading to an overestimation of TPC values [66,84].

During refrigerated storage, both DPPH and TPC activities significantly decreased (P < 0.05), with the lowest values observed at 28 days. When the combined effects of formulation and storage time were evaluated, no interaction effect was detected. The decrease in antioxidant content may be attributed to the degradation of phenolic compounds and to interactions between milk proteins and polyphenols, as well as to the metabolic activity of lactic acid bacteria during storage [14,85,86]

Additionally, chemical and enzymatic oxidation processes contribute to the loss of phenolic compounds, as these reducing agents react with oxygen and undergo degradation, which can be accelerated by traces of transition metals or residual oxidative enzymes [14,87]. In protein-rich matrices such as yogurt, the formation of insoluble complexes between polyphenols and caseins further reduces their ability to react with both the Folin–Ciocalteu reagent and the DPPH radical, leading to an apparent and functional decline in antioxidant values during storage [20].

Similar findings were reported by Kennas et al. [88], who reported higher antioxidant potential (DPPH) in yogurt enriched with pomegranate peel powder and honey, particularly in formulations containing higher concentrations of these ingredients (10% and 5%). The authors attributed this effect to the elevated phenolic content of these formulations.

Similarly, another study [89] reported that yogurt enriched with a relatively high honey proportion (5%) presented increased TPC compared with that of the control yogurt and reported a significant reduction in antioxidant activity after 28 days of storage, which is consistent with the observations of the present study.

### Technological properties of yogurt

#### Color, Syneresis and Determination of the WHC.

Differences (P > 0.05) were observed between treatments and storage days for all color parameters (L, a* and b*) (Table 4).

**Table 4:** Technological properties of yogurt with and without the addition of honey *A. mellifera* from the Amazon savanna at different concentrations over 28 days of refrigerated storage.

	Treatments					Storage							P < Value		
Analysis	CON^1^	H8%^2^	H12%^3^	H16%^4^	H20%^5^	1	3	7	14	21	28	SEM^6^	T^7^	D^8^	TxD^9^
Syneresis^10^	25.19^a^	25.56^a^	25.53^a^	24.26^a^	16.09^b^	22.41^B^	22.76^AB^	23.45^AB^	23.37^AB^	24.34^A^	24.04^A^	0.443	0.000	0.028	0.182
WHC^11^	51.31^e^	55.64^d^	58.68^c^	64.09^b^	78.82^a^	64.32^A^	62.78^AB^	62.53^AB^	61.30^B^	61.27^B^	58.04^C^	1.077	0.000	0.000	0.014
L*^12^	70.91^a^	69.70^a^	68.56^ab^	68.28^ab^	65.75^b^	62.85^B^	62.17^B^	72.63^A^	70.04^A^	72.64^A^	71.32^A^	0.607	0.00	0.000	0.180
a*^13^	2.06^b^	2.37^a^	2.37^a^	2.42^a^	2.44^a^	2.38^A^	2.37^A^	2.36^AB^	2.36^AB^	2.33^AB^	2.19^B^	0.023	0.00	0.019	0.092
b*^14^	4.96^d^	5.38^c^	5.63^bc^	5.85^b^	6.17^a^	4.91^B^	5.80^A^	5.87^A^	5.61^A^	5.66^A^	5.66^A^	0.059	0.00	0.000	0.916

Means of treatments with different lower-case letters in the same line are significantly different (P < 0.05). The means of storage with different uppercase letters are significantly different (P < 0.05).^1^CON – yogurt without bee honey; ^2^H8% – yogurt containing bee honey at 8% (v/v); ^3^H12% - yogurt containing bee honey at 12% (v/v); ^4^H16% - yogurt containing bee honey at 16% (v/v); ^5^H20% – yogurt containing bee honey at 20% (v/v). ^6^SEM: standard error of means; ^7^T = P value effect of treatment; ^8^D = P value effect days of storage; ^9^TxD = P value interaction between treatments and storage; ^10^Syneresis (%); ^11^WHC - water holding capacity (%). ^12^L*- measure of darkness to lightness (a higher value indicates a lighter color); ^13^a*- redness measurement (a higher value indicates a redder color)); ^14^b*- measure of yellow (a higher value indicates a more yellow color).

The luminosity (L*) of the yogurt decreased proportionally with increasing amounts of honey added. Compared with the H20% treatment, the CON and H8% treatments resulted in greater luminosity values. This finding can be attributed to the natural coloring compounds present in honey [6], which reduce the luminosity of yogurt as the concentration of honey increases. Similarly, Sert et al. [81] evaluated the influence of adding sunflower honey (2%, 4% and 6% w/v) to yogurt during cold storage. When both factors were evaluated simultaneously, no interaction was detected.

Similarly, Coskun & Dirican [72] reported a reduction in the brightness of yogurt with the addition of pine honey with increasing proportions of honey (2%, 4% and 6%), which was also observed in this study.

With increasing storage time, an increase in the L value (P > 0.05) was observed. Higher brightness values result in lighter objects, which may be associated with the characteristic white color of milk [6].

Regarding the parameter a*, differences were observed (P > 0.05), with the value of a* increasing with the concentration of honey in the yogurt formulation. The same behavior was observed for the parameter b* (P > 0.05). These results can be attributed to the yellow color of honey.

During cold storage, the a* parameter decreased significantly (P > 0.05), and the b* parameter showed inversely proportional behavior, increasing with the number of days of storage. This increase in the b* value can be explained by the destabilization of the gel structure with the release of serum, which is characterized by a greenish color [7]. The combined evaluation of both effects revealed no significant interaction.

When studying goat milk yogurt with honey and the probiotic *Lactobacillus acidophilus,* Machado et al. [6] reported similar changes in the color of yogurt at the end of the storage period and attributed this effect to the color of the added honey and the possible presence of compounds derived from the Maillard reaction.

Syneresis occurs when the yogurt gel loses the ability to retain the liquid phase, resulting in contraction of the protein network and subsequent whey separation. [31,90].

In the present study, yogurt formulated with 20% honey (H20%) presented significantly (P > 0.05) lower susceptibility to syneresis than the other yogurt samples did (Table 4), indicating an improvement in gel stability at higher honey concentrations. This reduction in syneresis can be attributed primarily to the high osmotic pressure and hygroscopic nature of honey, which promotes water retention within the casein network, limiting the migration of free whey [6].

Honey is composed predominantly of glucose and fructose, with fructose exhibiting superior water-binding capacity due to its molecular structure and strong interaction with water molecules. These characteristics favor the immobilization of water within the gel matrix, thereby reducing whey expulsion [81].

Additionally, the presence of phenolic compounds in honey may contribute to gel stabilization through noncovalent interactions with milk proteins, including hydrogen bonding and hydrophobic interactions [6]. These interactions can reinforce the three-dimensional protein network, increasing its resistance to contraction during storage and reducing syneresis. Similar behavior was reported by Ismail et al. [82], who reported reduced syneresis in goat milk dairy products supplemented with honey, particularly at relatively high inclusion levels.

In contrast, some studies have reported increased syneresis following honey addition, especially when low concentrations (≤6%) were used [81]. These discrepancies may be explained by insufficient honey levels to exert osmotic or structural effects, differences in honey botanical origin, phenolic profile, or variations in milk composition and processing conditions. Thus, the impact of honey on syneresis appears to be concentration-dependent and matrix specific.

The greatest degree of syneresis was observed on the last days of cold storage (Days 21 and 28) compared with the initial days (Days 1 and 3) (P > 0.05) because of the syneresis and rearrangement of proteins that occur during storage [31,91]. Analyzing both effects concurrently revealed no interaction. Whey separation, also known as whey-off, is a common defect of fermented dairy products during storage [1,92]. Segundo Saleh et al. [93] reported that this separation is caused by weakening of the casein network and mechanical damage to the gel structure.

Water holding capacity (WHC) is an indicator of the ability of a gel structure to retain serum [94] and is defined as the resistance to separation of the serum under applied force in high-speed centrifugation [68,95].

**Fig 2:**
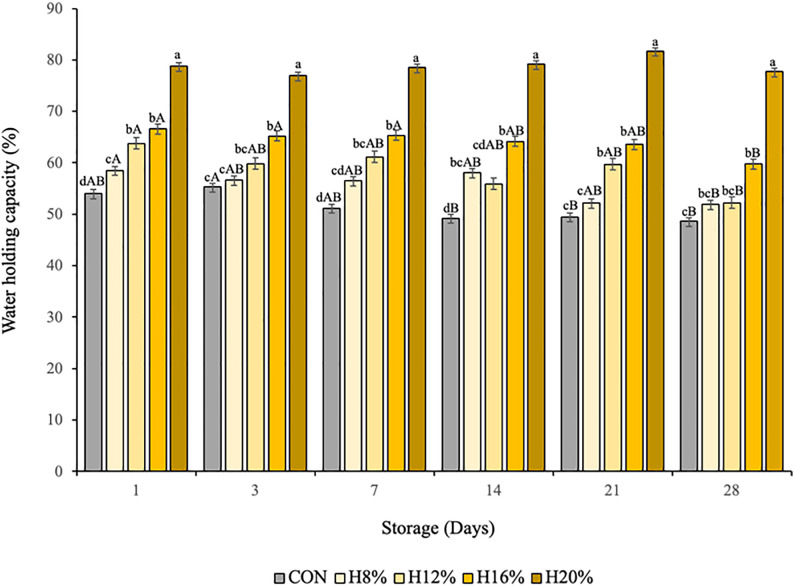
Interaction effect between treatment and days of storage on the WHC of yogurt with honey during storage. Different lowercase letters indicate significant differences between treatments on the same day of display (P < 0.050).^1^CON– yogurt without bee honey; ^2^H8% - yogurt containing bee honey at 8% (v/v); ^3^H12% - yogurt containing bee honey at 12% (v/v); ^4^H16% - yogurt containing bee honey at 16% (v/v); ^5^H20% – yogurt containing bee honey at 20% (v/v).

The WHC of yogurt was also affected by treatment and storage time (P < 0.05). CON yogurt (without honey) had the lowest WHC, while samples with honey had the highest WHC, in increasing order of honey concentration. The WHC was greater on the first day of storage than on the last day (28th day), indicating an interaction between treatment and storage time (Fig 2).

On the days of storage, the differences (P < 0.05) in the WHC among the treatments affected the WHC. Compared with CON yogurt (without honey), yogurt with the highest concentration of honey (H20%) presented the lowest water loss; notably, the WHC was greater when honey was added to the formulations. This is due to the lower degree of syneresis observed in these treatments. However, on the 14th day, the H12% treatment had a lower WHC than did the H8% treatment.

This behavior is related to syneresis, as water release was accompanied by a reduction in the WHC of the different yogurt samples during storage. This effect was also reported in other studies that evaluated the addition of honey to dairy products [6,82].

Differences (P < 0.05) were observed during storage for the CON, H8%, H12% and H16% treatments, with a reduction in WHC as the storage time increased. This suggests that the protein matrix of the gel is partially degraded during storage [95,96].

The CON yogurt (without honey) had a lower WHC, which indicates a weaker gel structure. Mohan et al. [95], when evaluating yogurt with different concentrations of honey and stored for 21 days, reported that the samples of yogurt sweetened with honey presented a greater WHC than the control yogurt did, with values ranging from 65% to 72%, and decreased slightly during the storage period.

### Microestructure

**Fig 3:**
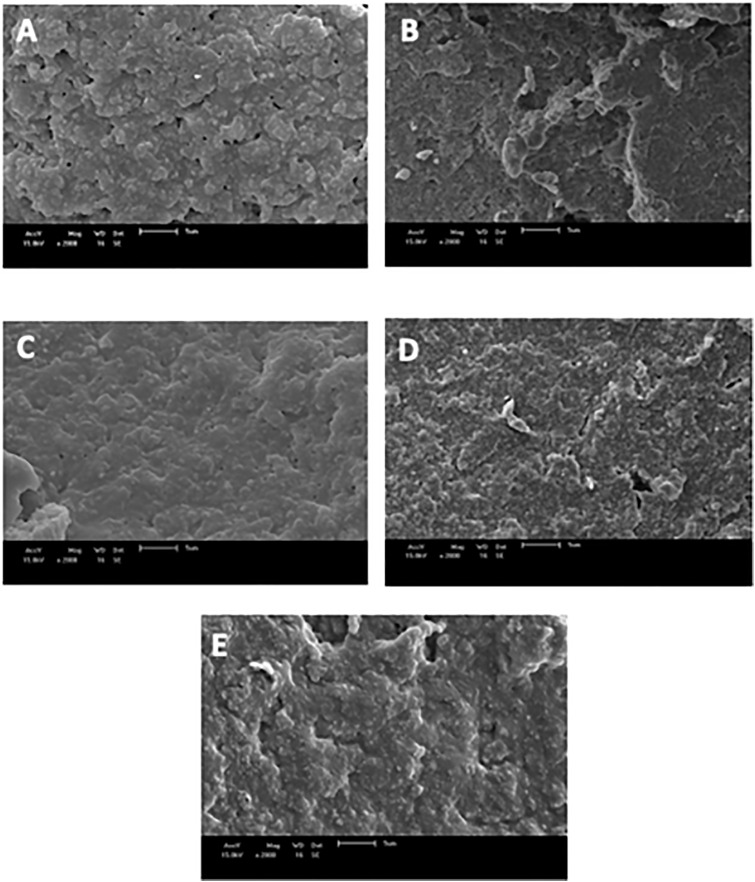
Scanning electron microscopy (SEM) images of yogurt supplemented with honey. CON **(A)**; H8% **(B)**; H12% **(C)**; H16% (D) and H20% **(E)**.

The microstructures of the yogurt samples were observed by scanning electron microscopy (Fig 3). All the yogurt samples presented a protein network with casein micelles interconnected with interspersed empty zones. In the CON (Fig 3-A) and H8% (Fig 3-B) yogurt samples, a relatively loose network structure was observed, with the agglomerates overlapping, whereas the H12%, H16% and H20% yogurt samples (Fig 3- C, D and E) presented a more compact gel network, with a more homogeneous and continuous network surface.

The voids observed in CON and H8% yogurt almost disappeared when the honey concentration was increased (H12%, H16% and H20%). Vital et al. [31] reported that networks with voids can promote contraction of the protein network and consequent structural rearrangement during storage, directly impacting the texture of the product. More homogeneous and compact surfaces result in less protein rearrangement, making yogurt softer and less susceptible to syneresis during storage [70].

### Sensory characteristics

The sensory analysis study was approved by the Research Ethics Committee involving human beings at the Federal University of Roraima (protocol number: 69320823.2.0000.5302), Boa Vista, State of Roraima, Brazil.

Sensory evaluations of the acceptance, purchase intention and general preferences of yogurts with different concentrations of *Apis mellifera* bee honey from the Amazon savanna are presented in Table 5.

**Table 5:** Consumer acceptability of yogurt containing different concentrations of *A. mellifera* bee honey from the Amazon savanna over 28 days of refrigerated storage (n = 134 consumers).

Variable	CON^1^	H8%^2^	H12%^3^	H16%^4^	H20%^5^	SEM^6^	*P<*
Color	7.78	7.64	7.80	7.71	7.67	0.044	0.073
Appearance	7.50	7.59	7.66	7.63	7.62	0.046	0.098
Aroma	6.80^b^	6.75b	7.07^ab^	7.02^ab^	7.40^a^	0.065	0.012
Flavor	7.27^a^	5.03^c^	6.62^b^	7.44^a^	7.66^a^	0.080	0.000
Texture	7.28^bc^	6.87^c^	7.49^ab^	7.56^ab^	7.81^a^	0.062	0.000
General	7.57^a^	5.73^c^	7.01^b^	7.31^ab^	7.66^a^	0.069	0.000
CI^7^	3.87^ab^	2.68^c^	3.57^b^	4.02^a^	4.13^a^	0.049	0.000
Preference	3.32^a^	1.99^c^	2.70^b^	3.40^a^	3.50^a^	0.055	0.000

Different lowercase letters on the same line indicate significant differences. ^1^CON – yogurt without bee honey; ^2^H8% - yogurt containing bee honey at 8% (v/v); ^3^H12% - yogurt containing bee honey at 12% (v/v); ^4^H16% - yogurt containing bee honey at 16% (v/v); ^5^H20% – yogurt containing bee honey at 20% (v/v); ^6^SEM: Standard error of means. Parameters were evaluated via a nine-point structured hedonic scale without a central point, ranging from 1: extremely disliked to 9: extremely liked; ^7 CI^ - purchase intention – assessed via a five-point unstructured hedonic scale, ranging from 5: would certainly buy to 1: certainly would not buy; Preference – assessed by choosing the most and least preferred sample, on the basis of the reference ranking from 1--5, with 1: least preferred and 5: most preferred.

The sensory evaluation revealed that there were no significant differences (P > 0.05) in color or appearance attributes. The sensory evaluation revealed that there were no significant differences (P > 0.05) in color or appearance attributes among the treatments, indicating that the incorporation of *Apis mellifera* honey from the Amazon savanna, even at relatively high concentrations (up to 20%), did not perceptibly alter the visual appearance of the yogurt. This result suggests that the pigments and natural compounds in honey are well incorporated into the dairy matrix, maintaining a homogeneous and appealing color for consumers [3].

On the other hand, significant differences (P < 0.05) were observed for the aroma, flavor, texture, overall acceptance, purchase intent, and preference attributes.

The yogurt containing 20% (H20%*) Apis mellifera* honey was highly accepted, with higher mean scores for aroma, flavor, texture, and purchase intent. The aroma scores increased gradually as the honey concentration increased, reaching the highest value in the formulation with 20% honey. This result may be associated with the presence of characteristic volatile compounds in honey, which positively contribute to sensory perception [17].

The flavor attribute showed more pronounced variation among samples, with the lowest value observed in the yogurt with 8% honey and the highest values in the formulations with 16% and 20% honey, which were statistically similar to those of the sucrose-sweetened control. The low acceptance of yogurt with 8% honey may be related to an imbalance between the natural acidity of the yogurt and the low sweetness level provided by the lower honey concentration. In contrast, the formulations with 16% and 20% contents presented a more balanced sweetness profile, better meeting consumer preferences.

These results corroborate the trend observed in the texture and overall acceptance attributes, which also showed the highest averages for samples with higher levels of honey incorporation [3,97]. When evaluating the acceptability of yogurt through sensory and analytical measures of sweetness and acidity, Barnes et al. [98] reported that consumers show a greater preference for sweetened yogurt, highlighting the strong hedonic appeal associated with sweet taste.

Machado et al. [6] reported that, in a previous study, probiotic goat milk yogurt (*Lactobacillus acidophilus*) with stingless bee honey presented significantly greater overall acceptability than did unsweetened controls, with formulations containing high concentrations of honey being the most preferred.

In addition to acting as a natural sweetener, honey may have contributed to a creamier and more pleasant texture because of the presence of reducing sugars and hygroscopic compounds that influence product viscosity [19,99].

Consumer intention (CI) and preference showed similar behavior to the other sensory attributes, with higher scores observed in formulations containing 16% and 20% honey. These findings corroborate the trend of greater acceptance of products with higher sweetness levels, a characteristic widely recognized in the sensory profile of the Brazilian consumer [6]. This pattern suggests that PI was strongly influenced by the perceived sweetness levels of the yogurt, indicating that the degree of sweetness plays a decisive role in the purchase decision and consumer preference [95].

In addition to the pleasant sensory profile, the perception of naturalness and the potential health benefits associated with honey may have contributed to the higher purchase intent and overall acceptance of yogurt with higher honey content [81,82]

Similar results were reported by Mohan et al. [95], who reported greater acceptance of yogurt sweetened with blends of Manuka honey, attributed to the positive consumer perception of sweetness and the functional benefits of honey.

## Conclusion

Honey from *Apis mellifera* bees originating from the Amazon savanna complies with national and international quality standards and presents relevant levels of phenolic compounds and antioxidant activity. When incorporated into yogurt formulations, this honey influences acidity during refrigerated storage and maintains consumer acceptance and preference. Formulations containing higher honey concentrations (16% and 20%) presented greater antioxidant activity and were more favorably evaluated by consumers.

Overall, the results support the technological potential of Amazon savanna honey as a natural ingredient for the development of differentiated yogurt formulations, particularly in terms of its physicochemical characteristics, antioxidant capacity, and sensory acceptance. Nevertheless, further research incorporating starter culture viability and additional functional parameters is necessary to provide a more comprehensive evaluation of product functionality.
